# Factors affecting the implementation of employee whole health in the veterans health administration: a qualitative evaluation

**DOI:** 10.1186/s12913-023-09450-3

**Published:** 2023-06-08

**Authors:** Omonyêlé L. Adjognon, Adena Cohen-Bearak, Jenesse Kaitz, Barbara G. Bokhour, Leslie Chatelain, Martin P. Charns, David C. Mohr

**Affiliations:** 1 Center for Healthcare Organization and Implementation Research, VA Boston Healthcare System, Boston, MA USA; 2 Department of Health Law, Policy & Management, Boston University School of Public Health, Boston, MA USA; 3 Center for Healthcare Organization and Implementation Research, VA Bedford Healthcare System , Bedford, MA USA; 4 VA Puget Sound Healthcare System, Seattle, WA, USA; 5 Department of Population and Quantitative Health Sciences, University of Massachusetts Medical School, Worcester, MA USA

**Keywords:** Health promotion, Occupational health, Holistic health, Change management, Employee wellness

## Abstract

**Background:**

There is increasing recognition of the need to focus on the health and well-being of healthcare employees given high rates of burnout and turnover. Employee wellness programs are effective at addressing these issues; however, participation in these programs is often a challenge and requires large scale organizational transformation. The Veterans Health Administration (VA) has begun to roll out their own employee wellness program—Employee Whole Health (EWH)—focused on the holistic needs of all employees. This evaluation’s goal was to use the Lean Enterprise Transformation (LET) model for organizational transformation to identify key factors—facilitators and barriers—affecting the implementation of VA EWH.

**Methods:**

This cross-sectional qualitative evaluation based on the action research model reflects on the organizational implementation of EWH. Semi-structured 60-minute phone interviews were conducted in February-April 2021 with 27 key informants (e.g., EWH coordinator, wellness/occupational health staff) knowledgeable about EWH implementation across 10 VA medical centers. Operational partner provided a list of potential participants, eligible because of their involvement in EWH implementation at their site. The interview guide was informed by the LET model. Interviews were recorded and professionally transcribed. Constant comparative review with a combination of a priori coding based on the model and emergent thematic analysis was used to identify themes from transcripts. Matrix analysis and rapid turnaround qualitative methods were used to identify cross-site factors to EWH implementation.

**Results:**

Eight common factors in the conceptual model were found to facilitate and/or hinder EWH implementation efforts: [1] EWH initiatives, [2] multilevel leadership support, [3] alignment, [4] integration, [5] employee engagement, [6] communication, [7] staffing, and [8] culture. An emergent factor was [9] the impact of the COVID-19 pandemic on EWH implementation.

**Conclusions:**

As VA expands its EWH cultural transformation nationwide, evaluation findings can (a) enable existing programs to address known implementation barriers, and (b) inform new sites to capitalize on known facilitators, anticipate and address barriers, and leverage evaluation recommendations through concerted implementation at the organization, process, and employee levels to jump-start their EWH program implementation.

## Background

The World Health Organization and the International Labour Organization (WHO/ILO) associate stressful workplace conditions and exposures (e.g., long working hours, gases, fumes and various chemicals) across fields and industries worldwide with employee adverse health outcomes (e.g., heart disease, depression, injuries and death), and call for employers to improve workplace conditions [1–3]. In US healthcare, workplace climate has been associated with burnout [4], with extremely high rates of burnout across healthcare professions (e.g., over half of physicians, and one-third of nurses) [5]. Burnout also has dire consequences for patient care (e.g., medical errors, patient satisfaction), and the healthcare system (e.g., cost of turnover) [4].

In response, leading US health institutions are promoting organizational change through workplace health [6], and an emphasis on whole person health, to empower individuals and communities to engage in the improvement of their health [7]. Employee wellness programs aligned with whole person health have the potential to decrease absenteeism and healthcare costs for employers and to encourage self-care, behavior and lifestyle changes, and improve employees’ overall health and wellbeing [8, 9]. Though many workplace wellness programs have been associated with reduction in stress, absenteeism, and burnout, while promoting sleep health, mindfulness and increasing employee resilience [10, 11], an integrated and holistic approach to employee wellness is recommended to optimize the productivity of healthcare workers and improve organizational competitiveness [12].

Prior work shows multilevel challenges to the implementation of wellness programs in the workplace. Organizational barriers include limited policy support for initiatives, lack of prioritization of employee health and wellbeing, lack of space, and lack of leadership and managers’ support [13–15]. Employee-level barriers to participation include logistics, such as staff schedule, perceived lack of time, and potential lack of interest in activities, with variable employee participation (e.g., 10–65% among registered nurses) [16, 17]. However, prior research on implementation of employee wellness programs in healthcare has not taken an organizational transformation perspective. This perspective recognizes the complex, multilevel nature of change, including culture change reflected in the organization’s values, expectations, and norms. Much of the empirical literature on organizational transformation in healthcare has addressed quality improvement [18], specifically Lean Management Systems [19, 20]. Although developed in quality improvement research, we are using the Lean Enterprise Transformation (LET) model [21], because it is a comprehensive model of transformation that includes constructs such as leadership, staff training and culture, for organizational transformation in general.

In recent years, the Veterans Health Administration (VA) — the U.S. largest integrated healthcare system including 171 medical centers — has implemented the Employee Whole Health (EWH) initiative focusing on employee wellness [22, 23]. Built on a Whole Health System of Care for patients that focuses care on patients’ overall well-being [24], EWH mirrors these goals by focusing on the well-being of the healthcare workforce.

This evaluation’s goal was to use the LET model for organizational transformation to identify key factors—facilitators and barriers—affecting the implementation of VA EWH.

## Evaluation methods

The Action Research Model for organizational change was the context for this study [25]. Action research is a reflective qualitative process that can be used within an organization to identify what changes need to be made, and results in the implementation of new processes [26]. Three cycles in action research include the initial change implementation (cycle 1), the first evaluation to revise and amend the implementation (cycle 2), and a repeat evaluation to further revise and amend the implementation (cycle 3) [26]. Our study focused on cycle 2, the first evaluation following the implementation of EWH.

### Evaluation design and setting

This is the first of a two-phase cross-sectional qualitative project to evaluate the organizational implementation of EWH across multiple sites in VA. Contextual factors identified through this study will help existing sites refine EWH implementation and inform future sites’ implementation of EWH. To design this evaluation, we adapted the LET model in which twelve constructs interact to enable any organization-wide transformation [21]. For example, in adapting the model, we replaced “communication about Lean” with “communication about EWH” (see Table 1). Qualitative interviews were designed to identify factors—barriers and facilitators—affecting the implementation of EWH.

**Table 1:** Sample interview questions by Lean Enterprise Transformation (LET) constructs regarding Employee Whole Health (EWH) implementation

	LET Construct	Definition	Sample Questions
1	Impetus for EWH implementation	Motivation behind EWH implementation	• Can you tell me about the history of how and when EWH initially got started at your site? • Are there other Whole Health programs for Veterans occurring at your site, and what are they?
2	Leadership commitment to EWH	Senior leaders and middle management engage in EWH, and participate in EWH activities	• What was the involvement of medical center leadership for EWH? • What was the attitude of service chiefs/service line managers and front-line supervisors regarding EWH? • To your knowledge, do leaders in this medical center participate in EWH themselves?
3	EWH implementation initiatives	Systematic organizational efforts to plan, schedule and conduct EWH activities	• What, specifically, is happening regarding EWH at your site? (Activities may include employee orientation training, other trainings, yoga classes, meditation, nutrition, coaching, Whole Health committee, etc.)
4	Alignment of EWH across the organization	Leaders across levels and departments encourage involvement in EWH, and provide resources (e.g., protected time, equipment) for EWH implementation	• Did the implementation team or executive leadership have a strategy for implementing EWH? • What did leaders do to support the implementation of EWH? • Do employees believe that they can take time to participate in EWH during working hours guilt-free, or with their supervisors’ approval?
5	Integration of EWH across internal boundaries	Staff work together effectively and willingly across department and service lines	• Did your organization try to implement EWH across the whole medical center at once or start in a particular area of the medical center? Are there areas/departments or services where this has really taken off? • Are there other services within the medical center that the EWH initiative collaborates with or is dependent upon?
6	Communication around EWH	Communication tools and actions to raise awareness around EWH within, across and outside the organization	• How is EWH information communicated? • Is there regular communication to employees about Employee Whole Health? • Does your site have a formal communication plan?
7	EWH knowledge and skills	Organization help staff build EWH knowledge and skills; staff use these to improve health/life balance and overall well-being	• Have you heard from participants that EWH has made a personal difference? • To your knowledge, has any of them shared their experiences with other co-workers?
8	Informed decision-making	Meaningful data regarding key EWH activities is collected and tracked to inform EWH implementation progress	• Do you track or monitor data on EWH activities?
9	Employee/staff engagement	Employees’ feedback is received and used as part of EWH implementation	• How receptive do front-line employees seem to be about EWH implementation?
10	Organization culture	What people – not only managers but also staff–value and expect as being appropriate EWH behavior	• Do you think the culture of this medical center values participation in EWH activities? • Does perception of EWH vary by different parts of the medical center?
11	Staffing	Staffing levels and skills are adequate for EWH implementation	• What resources does your medical center provide for Employee Whole Health implementation? • Who else is on your EWH implementation team? (their roles and how you interact with them)
12	Using EWH experts	EWH experts are solicited and transfer key knowledge to sites’ EWH implementation team	• What was the involvement of the national program office? • Is your site working in partnership or collaboration with your VISN for EWH operations?

Note: LET = Lean Enterprise Transformation; EWH = Employee Whole Health; VISN = Veterans Integrated Service Network

The VA Office of Patient-Centered Care and Cultural Transformation (OPCC&CT) funded medical centers nationwide to embark on the Whole Health cultural transformation journey starting with 18 “flagship” medical centers in 2017 and continuing with 37 additional medical centers in 2019–2020 [23, 27]. These 37 medical centers could select among three foci: clinical care delivery, peer-support, or EWH. This evaluation’s goal is to conduct a cross-site qualitative evaluation of sites that selected EWH program implementation as a focus. The study was reviewed and classified by VA Bedford Healthcare System and VA Boston Healthcare System Institutional Review Boards as a non-research operations program evaluation. Thereafter, the Research and Development Committee provided oversight for the work. All methods were carried out in accordance with relevant regulations. Informed consent was obtained from each participant.

### Site selection

Between March 2019 and January 2020, ten medical centers included EWH in their Whole Health transformation effort. As a result, these sites participated in OPCC&CT-led EWH training sessions including a face-to-face kick-off meeting and subsequent phone calls from content experts. All ten of these ten sites were included in the evaluation.

### Sample of participants

We used purposive sampling to identify key informants—most knowledgeable about EWH implementation— eligible to participate in the study [28]. To collect data from multiple perspectives, we aimed to interview two to three key informants in various roles at each of the ten sites. Key informants from each site included EWH core implementation team members (e.g., EWH coordinator), at-large Whole Health staff (for sites with more than one Whole Health focus), and sites’ wellness and occupational health staff who supported the EWH efforts (see Table 2). OPCC&CT provided the evaluation team with EWH staff information (name, role, and email) at eligible sites. The evaluation team contacted potential participants individually through emails for voluntary and confidential participation in the study. Individual phone interviews were scheduled with key informants and their verbal consent was obtained for the interviews. The evaluation team had no relationship with participants prior to recruitment for data collection.

**Table 2:** Key informant roles

Key informant roles	n
WH/health promotion program manager	9
Health coordination or health coaching	5
EWH program manager	4
Leadership (senior and mid-level)	3
Education support	3
Program assistant	3
Total	27

#### Eligibility criteria

All staff on the provided list were eligible to participate because they have been involved in EWH implementation at their site and as such qualified as key informants. We invited 29 eligible potential participants, and only two declined because they had changed roles and were no longer involved in EWH implementation at the eligible sites.

### Data collection

#### Semi-structured interview guide

We developed an interview guide containing open-ended questions from the LET constructs. We pilot tested the guide by interviewing three key informants from sites outside of our sample and refined the guide accordingly. Our final interview guide (sampled in Table 1) contained questions in 12 of the model’s 13 constructs (e.g., leadership commitment to EWH, alignment across the organization, communication, organization culture, staffing). We eliminated questions about patient/Veteran engagement because in pilot testing, we found this was not relevant to EWH. In addition, we asked participants to identify their perceived biggest barriers and facilitators to EWH implementation, and suggestions for improving implementation.

#### Interview procedure

We conducted individual 60-minute, semi-structured interviews with participants from February 2021 through April 2021 through Microsoft Teams software, a live video conference service. Three investigators, ACB, OLA and JK, conducted the interviews. Interviews were recorded with participants’ permission and assigned unique identifiers to protect participants’ privacy and confidentiality. To reduce interviewer bias, we used the semi-structured interview guide described above to conduct all interviews. In addition, each interviewer completed debrief notes at the end of each interview using an excel template. Debrief notes were later compared with matching transcript data for accuracy. De-identified recordings were professionally transcribed using institutionally approved procedures.

### Data analysis

Data analysis combined the Miles and Huberman framework for qualitative data analysis (i.e., data reduction, display and conclusions) [29] and matrix analysis for rapid turn-around of qualitative findings in implementation research [30]. In addition, we used a constant comparative review process with a combination of a priori and emergent codes to identify themes from the transcribed interviews [31]. All evaluation team members—highly experienced in qualitative methods and in organizational transformation—reviewed two transcripts to develop a common understanding of recommended coding practices. Thereafter, teams of two independently coded the same interview, recording responses in a template organized by constructs. For each construct, the template included the following sub-sections: (a) description, (b) facilitators, (c) barriers, and (d) key quotes. Three team members, ACB, OLA and DM, then reviewed and compared the two codes per transcript for consistency. Thereafter, the two team members met to discuss and reach consensus as needed. This process was repeated for each of the remaining transcripts. After all interviews had been coded, reviewed, and the responses consolidated in the template, we created a site-specific profile of barriers and facilitators for each site. Finally, we created a cross-site matrix to review and identify common themes reported across sites.

## Results

### Participant characteristics

Across the ten sites, we conducted three interviews at each site, with two exceptions: due to EWH staffing shortages there were only two people to interview at one site and one at another site.

### Factors affecting EWH implementation

As summarized in Table 3, findings from our analysis highlight 9 most common factors (i.e., discussed by at least four sites) affecting EWH implementation, either by facilitating and/or by posing challenges to EWH activities and engagement. Eight factors are constructs from the adapted LET model [28]: (1) EWH initiatives, (2) multilevel leadership support, (3) alignment, (4) integration, (5) employee engagement, (6) communication, (7) staffing, and (8) culture. One emergent factor is (9) the impact of the COVID-19 pandemic. Table 4 presents illustrative quotes for each factor.

**Table 3:** Summary of factors affecting EWH implementation by construct

1. Key factors by construct from the extended Lean Enterprise Transformation model		
Construct	Facilitator	Barrier
EWH initiatives	Sites implemented a variety of EWH activities in various formats to engage staff	Sites faced challenges to reach specific groups of employees
Multilevel leadership support	Senior leadership • Provided clear directives for and engaged in strategic planning for EWH • Personally took part in EWH activities Middle management • Understood the importance of employee self-care • Allowed time for staff to engage in EWH activities Program leadership • Drive for results helped achieve program goals • Champions engaged staff by making connections across services • Worked in well-functioning EWH committees	Senior leadership • Did not support giving staff protected time to engage in EWH activities • Turnover at times decreased the level of support for EWH implementation Middle management • Support was not uniform across services within a medical center • Limited or denied employees protected time to engage in EWH • Concerns for staff productivity led to lack of support for EWH Program leadership • Uncertainty about EWH’s place in the organizational structure • Struggles to advocate effectively for program in the absence of formal EWH committee to guide implementation efforts
Alignment	• Leadership facilitated the acquisition of equipment • Leadership provided staff protected time for EWH • Dedicated physical space	• Leadership either denied or prevented resource provision • Lack of protected time prevented staff from engaging in EWH activities • Inadequate space for EWH activities
Integration	• Networks provided direct advocacy and support for local EWH programs • Partnerships/collaborations across services within sites helped with communication and increased awareness	• Perception that networks had little to no direct involvement with local EWH programs • Lack of collaboration between services at some sites hindered engagement in EWH activities
Employee engagement	Employees • Understand the importance of health and well-being • Interest in, enthusiasm for, and receptivity regarding EWH activities • Testimonials, word of mouth and positive feedback Spread of EWH • Synergies across services and departments • Focus on specific services or workgroups	Employees • Religious beliefs preventing participation in EWH activities • Not being used to self-care • Reluctance in the absence of clear guidance for participation • Fear of retaliation or to be misperceived by managers • Conflicts with timing of sessions offering (e.g., after working hours) Spread of EWH • Uneven opportunities to engage in EHW for some services • Lack of awareness or exposure in specific services (e.g., canteen staff)
Communication	• Technology helped build communities and made leadership direct interaction on platforms visible • Clear and consistent messages from senior leadership promote EWH • Inclusion of EWH in new employee orientation and the use of flyers and printed materials at events helped raise awareness and interest in EWH	• Some employees disliked mass emails • Employees who were not added to EWH-specific group lists didn’t receive the information on EWH activities • Limited access to communication tools prevented wider scale information-sharing
Staffing	EWH implementation team • Staff consistency and longer tenure contributes to efficient work • Timely hiring of team members with the right skillsets was valuable • Having a Whole Health department leveraged human resources for EWH	EWH implementation team • Missing key roles due to slow hiring process or site-imposed limitations • Understaffing (e.g., split positions, staff detailed due to COVID-19) • Loss of key implementation roles disrupted established processes that they managed Employees • Staff with heavy workload due to understaffing could not engage in EWH activities
Culture	• Employee-positive culture on self-care motivated employees • Newer staff in sites with mixed culture were enthusiastic about EWH	• Staff with longevity at sites with mixed culture did not embrace EWH • Being in a state or community where culture did not prioritize health • Being at a site where the culture prioritized work duties above all else
2. **Emergent factor**		
Construct	Facilitator	Barrier
Impact of COVID-19 pandemic	• Moving to virtual platforms allowed for inclusion of larger workforce segments • Increased interest in using EWH to help address employees’ burnout	• Limits placed on the types of EWH activities to maintain safety • Human resources were mobilized to address the pandemic and diverted from the EWH program • Shifting priorities reduced traction for EWH, and overall resources made available for EWH activities

**Table 4:** Exemplar quotes by construct

Construct	Exemplar Quotes
1. EWH initiatives	**Facilitator**
	“I think that about where we found our sweet spot was when we started implementing these sessions (lunch and learn) because then it all started to come together and so it – it started off slow and then we just took off.” (site 2)
	“My team from the Department, they were all like, “Let’s do that as much as we can,” ‘cause, we deal with complicated patients, and we need a release, and we’re working 10-hour days, and we never give ourselves that time. Even that 15 minutes of time is just -- it just makes a world of difference just to release some of that pent-up energy. And, so, it’s very well received, very much appreciated.” (Site 9)
	**Barrier**
	“Being a tertiary Mental Health Clinic, we’ve just got a ton of licensed people running around, and all of their ethical practice guidelines do not allow for engaging in dual relationships. And, so, utilizing a shared gym area with the Veterans we serve, having employees do that with the Veterans, could potentially constitute an environment of dual relationships, and, so, we cannot do that. Just too many licensed people.” (Site 10)
	“I think the biggest challenge that we have is how do we access those people who do not work in front of a computer… employees who are working in housekeeping, the people who are working in the cafeteria and basically, they work nonstop, and they get two 15-minute breaks, and they get a 30-minute lunch…That’s the part of the workforce that we don’t have a plan for yet.” (Site 1)
2. Multilevel leadership support	**Facilitator**
	“I’ve had complete support…We have our medical center director kind of lead opening remarks, that type thing. So very much hands-on in that respect.” (Site 2)
	“So the supervisor…they’ve actually have helped us with recruiting by sending emails out themselves and they’ve taken some of the courses themselves so that way they know what is going on and they can actually get those same skills that their supervisors and employees are getting.” (Site 1)
	“When you see your leaders participating, I think that sends a really big message that it’s okay to participate in certain things…I have been pleasantly surprised at how many of our service chiefs have been involved. Sometimes our service chiefs are teaching the classes.” (Site 3)
	**Barrier**
	“One of the biggest things is none of our leadership has even come to any Whole Health opportunity, ever. I mean that is the first, like, easiest step to me -- to at least come to something, participate in something, and show some type of interest.” (Site 8)
3. Alignment	**Facilitator**
	“The game changer was the [Learning Collaborative 2] funding. I mean that really made the big difference for us and it just allowed us to decide whether we wanted a fulltime employee heading Employee Whole Health and we already knew we wanted to do that before we got the money.” (Site 9)
	**Barrier**
	“We really need some time for our providers or clinicians, all of our staff to be able to participate in these programs and then to really understand what it’s about to make those fundamental changes…. So, I think there have to be some systemic changes. So, yes, we will need funding. We will need personnel, and we need time.” (Site 9)
4. Integration of EWH	**Facilitator**
	“We do outreach to new employees.…This is an email or a phone call. …In our core team, we do feel it is important to educate new employees early because really Whole Health is something that we hope to have them bring not just to their employee journey, but their experience with the Veterans as well. So, it’s better to set the bar early than to kind of change a behavior later.” (Site 8)
	**Barrier**
	“So, supervisors understood that their staff needed some kind of support, but at the same time there was like a real anxiety around workload, and I think…trying to find that balance, for supervisors, was really -- I think that was probably really hard for them.” (Site 5) “I think sometimes the politics are present and…I feel like the way they’ve divided things up just creates more silos and that there’s a lot of overlap between things and like if people that are working on Whole Health within the Behavioral Health service line don’t know what leadership has planned or what’s going on in Employee Wellness… I just find it very frustrating.” (Site 6)
5. Employee engagement	**Facilitator**
	“I’ve had people stop me in the hallway and say, ‘hey, you know, we were encouraged by seeing everybody out there and I’m trying to do, you know, I’ve got some wellness goals and things of that nature and people share those with me, which I think is really neat.” (Site 7)
	“You find these people that want to be champions, and then you work with them and then that way their departments get engaged, whether that was a formal agreement or not at the beginning…Our Whole Health [team member]… she’s developed a following during her Thriving Thursday series because she’s able, … I have employees who are just terrific and they can identify what they wanna teach to their fellow coworkers and I find that – for their own job site satisfaction, it makes a huge difference.” (Site 1)
	**Barrier**
	“I think that’s because they are afraid of, you know, feeling guilty or getting blamed for something or get in trouble because we don’t have the executive leadership saying, yes this is protective time, this is okay if you wanna go, we approve it…if we could get that I think that would really change a lot of things.” (Site 5)
	“It depends on the service…the PACT teams just feel buried and they don’t – if they – if they have a few extra minutes, they’re not gonna get their mindfulness call, they’re gonna go take lunch.” (Site 9)
6. Communication	**Facilitator**
	“I work in Employee Occupational Wellness and a lot of people come through…every interaction is a Whole Health opportunity” (Site 6)
	“I work with our Public Affairs officer, and she’s been wonderful to work with and help us get information out in that weekly missive and then also keep us connected with opportunities that might come from other areas around the state and other communities, and how we can engage employees that work in those communities in those opportunities.” (Site 10)
	**Barrier**
	“Our team very much frowns on the idea of a lot of this stuff … I think it’s an argument over beautification versus information. It’s one thing whenever we put up posters and signs, and it may be great to get this information out there but if those responsible are not coming around and taking it down afterwards, it causes squatter to build and so they very quickly said, no, no, no, no we’re not allowing this anymore.” (Site 2)
	“Our poor program assistant just got some really nasty emails back from staff about “stop emailing me this crap,” you know, “I don’t appreciate it. You’re bogging down my email. I don’t need this stuff. I don’t have time for this. Stop sending me this.” Like, there was just a lot of negativity sent to her when she was just simply the messenger.” (Site 8)
7. Staffing	**Facilitator**
	“[EWH program manager] could hit the ground running and had a lot of things already in her toolbox.” (Site 9)
	“Last year we got our first Whole Health [full time equivalent employee]. We have a [Patient Services Assistant] and she is really the backbone now for everything Whole Health. It’s not just Employee Whole Health, it’s Whole Health for the whole facility.” (Site 6)
	**Barrier**
	“The Whole Health partners are imbedded in the services and you – and you have the coordinator that has a split duty doing something else as well.” (Site 3)
	“I think the staffing issue in particular is to be noted because if you have an extreme shortage of staffing, not only are those people working very feverishly to take care of Veterans, but it would also be perceived very poorly if they were to engage in wellness activities.” (Site 2)
	“I can say personally as a provider working in Whole Health for almost three years now, I’m extremely burnt out of Whole Health, just from just a lack of support and just knowing that, yes, there are people that are divinely interested in Whole Health and do talk the talk and walk the walk, but there are so few of us that we’re so dulled that our flames are, like, out.” (Site 8)
	“So, supervisors were just like itching for something to offer staff ‘cause they knew. Like, they understood that their staff needed some kind of support, but at the same time there was like a real anxiety around workload and I think…tryin’ to find that balance, for supervisors, was really -- I think that was probably really hard for them.” (Site 5)
8. Culture	**Facilitator**
	“People are encouraged to take their lunch break and to get outside on their lunch break. We have a beautiful campus… When I would go outside to eat lunch or something on a picnic table, people were out there walking… nurses in their scrubs going for a quick walk, and so it just really is a culture at the medical center that continues to become the norm to take care of yourself and to take a break and get some exercise even if it’s a short walk, it just helps… Five years ago, you’d never seen people out walking on their lunch break…So, to see that transition, I – it’s just really nice.” (Site 7)
	“There was a staff member that attended a session, that was like ‘this is what gets me through.’ …Wednesdays are a hard day for their clinic. And she shared with us this is what gets her through the second half of her day. So, being able to have protected time where your -- you know, it’s one thing to get up and walk away from your computer…. You have that protected time for self-care. So, she said that she couldn’t get through her Wednesdays without that.” (Site 8)
	**Barrier**
	“A lot of our administrative staff feel a little bit more free to participate in Whole Health activities or just getting outdoors and say going for a ten-minute walk on a break. Whereas medical or mental health units where their task of being there to provide care or in our outpatient clinics, it’s a little bit more challenging. It’s like they don’t have the same sense of freedom to be able to participate like you see in the administrative areas.” (Site 10)
9. The impact of COVID-19	**Facilitator**
	“I know COVID has caused us a lot of grief but in a lot of ways it’s felt to open our eyes to other possibilities and so, … we’ve continued these Lunch and Learns. There’s no reason why we can’t continue that ‘cause employees love it.” (Site 2)
	**Barrier**
	“So, with COVID all that stuff had to stop in-person and it just seems like it’s been a little bit of a struggle with like upper management to allow us to have time to set up, you know, the [VA Video Connect] or Teams links, you know, to offer the classes.” (Site 5)

#### EWH initiatives

Sites implemented many EWH activities, including in-person, group or individual sessions (e.g., yoga) and virtual sessions (e.g., mindful moments, remote nutrition sessions). A participant noted their team’s enthusiasm with the EWH initiative:

*“My team from the Department, they were all like, “Let’s do that as much as we can,” ‘cause, we deal with complicated patients, and we need a release, and we’re working 10-hour days, and we never give ourselves that time. Even that 15 minutes of time—it just makes a world of difference just to release some of that pent-up energy. And, so, it’s very well received, very much appreciated.”* (Site 9)

Many sites used the VA’s online training system to deliver EWH content. Some sites noted that supervisors were more supportive of virtual employee involvement where staff could attend EWH activities without having to leave their work area. However, sites faced challenges either to reach employees without access to computers (e.g., housekeeping and kitchen staff), or to provide a timely response (e.g., sending link to participate) to staff who sign up for sessions in the online training system minutes before sessions start.

In addition, many sites acknowledged the limitations with EWH offerings both in terms of timing (e.g., offered only during lunch break or after working hours) and variety of activities. Some sites tried to address the issue by recording sessions for viewing later, or by diversifying topics and times of activities. One site’s lack of resources prevented regular scheduling of sessions, such that EWH activities were sporadic. A participant observed:

*“I think the biggest challenge that we have is how do we access those people who do not work in front of a computer… employees who are working in housekeeping, the people who are working in the cafeteria and basically, they work nonstop, and they get two 15-minute breaks, and they get a 30-minute lunch…That’s the part of the workforce that we don’t have a plan for yet.”* (Site 1)

#### Multilevel leadership support

Leadership was mentioned often, including in combination with how leadership affected other themes. Sites discussed the value of senior and middle management leadership support for EWH implementation, in both their demonstrated values and actions. In addition, program leadership played a key role in EWH implementation.

##### Senior and middle management leadership

Sites made implementation progress when they had senior leaders who believed in the mission of EWH and understood the importance of promoting employee psychological and physical health. Supportive senior leaders provided clear directives based on the national EWH framework, engaged in the medical center-level councils and EWH planning committees, and/or assisted in the creation of the EWH strategic plan. Some senior leaders also engaged through personal participation in EWH, including holding mindfulness sessions during town hall meetings, walking, or running a marathon. This engagement from senior leadership signaled the importance of EWH to all. Sites also discussed how middle managers talked with their colleagues about the importance of having employees take care of themselves. As a result, supervisors increased their willingness to grant administrative leave time for frontline staff and to integrate Whole Health practices into clinical care activities. Middle managers who valued self-care for employees saw EWH as a way to address stress and employee turnover and supported EWH implementation. In a participant’s words:

*“When you see your leaders participating, I think that sends a really big message that it’s okay to participate in certain things...I have been pleasantly surprised at how many of our service chiefs have been involved. Sometimes our service chiefs are teaching the classes.”* (Site 3)

Some sites were more challenged with limited support from leadership. EWH activities could only be offered before work, during lunch breaks or after work at sites where senior leadership did not support providing staff protected time for EWH during work hours. Also, senior leadership were unavailable to meet and learn more about EWH despite the program leaders’ repeated attempts. Lastly, departures of senior leaders (e.g., assistant director, chief of staff) who had been strongly supportive of EWH changed the level of support at a few sites, as new leaders had different priorities than their predecessors. In an extreme case, a new senior leader stopped all EWH activities and dissolved the EWH committee.

*“One of the biggest things is none of our leadership has even come to any Whole Health opportunity, ever. I mean that is the first, like, easiest step to me -- to at least come to something, participate in something, and show some type of interest.”* (Site 8)

At the middle management level, support was not uniform throughout medical centers for many sites. For instance, middle managers were publicly divided in their support of EWH at a site. At many sites, supervisors and managers either did not afford employees protected time to attend EWH events or limited it during working hours (e.g., weekly morning Grand Rounds or noon Lunch ‘N Learn). Last, many sites mentioned that middle management in some departments were not supportive of EWH due to concerns that employees will miss work for EWH activities. They perceived time spent in EWH as competing against patient care.

##### Program Leadership

Program leadership—through champions and EWH committees—was also a key factor for EWH implementation. First, many sites noted that EWH program goals could be achieved by identifying people—formal (e.g., EWH program manager) and developing committees or integrating EWH into existing committees. Committee members were drawn from different departments, which allowed them to spread the message of EWH. An overlap between members of related committees (e.g., Veterans WH and EWH) was noted as supporting the goals of the program at some sites. Sites found well-functioning EWH committees (i.e., with regular meetings to discuss program operation) helpful to EWH implementation.

Sites without a formal EWH committee to guide implementation struggled to effectively advocate for and encourage EWH. For instance, there was often uncertainty about EWH’s place in the organizational structure, and whether EWH should be in an existing department/service or stand-alone in the organization. Without a committee to discuss the program positioning, EWH was being implemented with an unclear structure at these sites.

A second approach to program leadership involved informal champions (e.g., clinical staff). These are people who [1] are passionate about EWH, [2] can take the EWH program message to the field to encourage and motivate staff and coworkers to “have fun with getting healthy,” and [3] are interested in sharing their talents with other employees through teaching courses or offering Whole Health-related services. Several sites also highlighted that champions significantly contributed to employees’ engagement by making connections within and beyond their service areas or departments. Champions were often speakers at events and discussed EWH in positive ways.

#### Alignment

A central element of alignment is the provision of resources instrumental for EWH. Sites discussed various tangible resources (e.g., products and services, human, financial), protected time, and physical space.

First, leadership facilitated acquisition of equipment, such as pedal exercisers which are used while seated at desks, mindful moment affirmation practice cards, yoga mats, aromatherapy, and membership access to the Ohmpractice online service for employees. These resources helped to cement EWH at these sites. One site remodeled the fitness center and purchased equipment and licenses for Whole Health materials with funding from their regional network of medical centers (known as VISN). Additional resources mentioned included various kits (e.g., a fitness-in-a-box kit being sent to community-based outpatient clinics, making Power of Habit kits available to interested staff), and snacks and refreshment to encourage attendance at events. Funding for dedicated EWH staff was also critical to implementation.

*“The game changer was the [Learning Collaborative 2] funding. I mean that really made the big difference for us and it just allowed us to decide whether we wanted a fulltime employee heading Employee Whole Health and we already knew we wanted to do that before we got the money.”* (Site 9)

Yet, in some sites, senior leadership prevented or denied resource provision for EWH implementation either by not supporting grant applications for EWH funding, not providing adequate staffing, or denying approval for hiring needed positions (e.g., WH Program Manager) despite available funds. These resource barriers hindered EWH implementation.

Second, medical center leadership at a few sites provided protected EWH time for employees as part of a collaborative effort with the national program office, yet many employees were not aware of or unable to use that protected time for EWH. One site piloted offering nurses one hour per week to use for WH activities. At other sites, the lack of protected time dedicated to EWH activities prevented staff, especially clinical staff, from engaging in EWH.

*“We really need some time for our providers or clinicians, all of our staff to be able to participate in these programs and then to really understand what it’s about to make those fundamental changes…. So, I think there have to be some systemic changes. So, yes, we will need funding. We will need personnel, and we need time.”* (Site 9)

Many employees wondered if they could use leave or compensatory time because it was unclear if they could get supervisory approval for EWH during work hours. Some reported that they signed up for a training in the past but were not allowed to attend, or that misunderstandings regarding engagement in EWH led to union involvement.

Last, dedicated physical space was another key resource for EWH. Specific examples of this resource provision included building a space for a WH department to include a gym with treadmills and elliptical machines, opening an outdoor gym area, and creating relaxation rooms and walking paths. However, a few sites reported inadequate space to carry out EWH activities such as yoga, tai chi, healthy kitchen classes, and gym use. Smaller facilities, such as the community-based outpatient clinics were especially challenged for space to conduct EWH activities. A site described how many programs competed for the same space, and not securing that space led to EWH program closure despite adequate funding. Because space is a scarce resource, senior leaders had to make commitments for space for EWH activities, and this did not occur when senior leaders did not value and prioritize EWH.

#### Integration

Multilevel partnerships and collaborations were important to promote EWH, but challenges they faced were detrimental to EWH implementation. These partnerships and collaborations formed between sites and their VISNs (regional networks), and between departments/services within their medical centers.

Sites mentioned that VISNs supported their programs either by expanding activity offerings and education, or through direct advocacy. Some sites noted how shared calendars with other medical centers within the VISN gave employees access to more times for participation in EWH activities and a variety of course offerings. A few sites discussed how VISN-level education calls and activities allowed the site to see what other medical centers were doing, particularly around the areas of developing leadership support, hiring practices, and sharing resources for not having to “re-invent the wheel.” In addition, many sites mentioned general advocacy from the VISN (e.g., through VISN-level steering committee) that created a supportive environment. They also mentioned specific teams or individuals, such as a VISN-level nurse or director who had advocated for EWH. Nonetheless, many sites perceived little to no direct VISN involvement in their site-specific EWH program. They believed this reluctance from the VISN was due to facilities being too different from one another in key focus areas and approaches to EWH implementation.

Within medical centers, sites reported benefiting from developing partnerships or collaborations with Public Affairs and Medical Media in addition to various other service areas or departments. First, these collaborations helped with communications and announcement of key activities which led to greater awareness of the EWH message among employees. An example:

*“We do outreach to new employees…This is an email or a phone call…In our core team, we do feel it is important to educate new employees early because really Whole Health is something that we hope to have them bring not just to their employee journey, but their experience with the Veterans as well. So, it’s better to set the bar early than to kind of change a behavior later.”* (Site 8)

Second, most sites described specific clinical service areas or departments, commonly primary care or mental health, that were involved in promoting EWH. These partnerships were often born from individuals in these service areas or departments who were interested and engaged in teaching WH to their peers. Departments and services offered specific help through staffing wellness fairs, offering short massages after employee walks, producing posters and materials, and spreading the message within their service area to other employees. In contrast, silos at some sites prevented some departments from collaborating and engaging in EWH activities.

*“I think sometimes the politics are present and…I feel like the way they’ve divided things up just creates more silos and that there’s a lot of overlap between things and like if people that are working on Whole Health within the Behavioral Health service line don’t know what leadership has planned or what’s going on in Employee Wellness… I just find it very frustrating.”* (Site 6)

#### Employee engagement

While EWH initiatives focused on program reach, employee engagement focused on program users, staff or employees. Most sites mentioned that many employees having a natural inclination and interest in EWH helped with program engagement. Many employees had a “good understanding of the importance of health and well-being.” Other examples included general enthusiasm of frontline staff, employees’ receptivity to different types of sessions, and staff volunteering for EWH planning and events. Lastly, employees voicing support through testimonials and word of mouth and having positive things to stay about the program, especially about training sessions they attended piqued peers’ curiosity to attend EWH events.

*“I’ve had people stop me in the hallway and say, ‘hey, you know, we were encouraged by seeing everybody out there and I’m trying to do, you know, I’ve got some wellness goals and things of that nature and people share those with me, which I think is really neat.”* (Site 7)

However, staff perceptions and attitudes were barriers to employee engagement and negatively affected implementation. Many sites reported that some employees refused or hesitated to engage in EWH due to viewing tai chi or yoga as contrary to their own religious beliefs, or staff were unfamiliar with practicing self-care, or simply lack of interest. A few sites noted that non-clinical staff had less perceived freedom or opportunity to participate than clinical staff. In addition, sites reported employees’ reluctance to engage in EWH in the absence of clear policies or guidance for participation (e.g., having protected time). While some employees didn’t want to be perceived as avoiding work, others feared retaliation from unsupportive managers for participating in EWH, especially when it was unclear if senior leadership at their site was onboard.

*“I think that’s because they are afraid of, you know, feeling guilty or getting blamed for something or get in trouble because we don’t have the executive leadership saying, yes this is protective time, this is okay if you wanna go, we approve it… if we could get that I think that would really change a lot of things.”* (Site 5)

A few sites highlighted that scheduling limitations and staff access to information negatively affected employee engagement in EWH. For example, at one site, EWH sessions were offered only after working hours to abide by federal regulations as this site understood them.

Sites took several approaches to spread engagement in EWH. One strategy aimed for facility coverage through synergies, by building and establishing rapport across different departments and services, which was easier with smaller facilities. Another strategy dispersed WH staff and clinical champions throughout the facility to create a collaborative network for spread. A third strategy focused on specific services or workgroups for initial EWH implementation. While one site with nursing leadership support identified nursing as the service for initial EWH implementation, another site used results from their annual all employee survey to identify workgroups with lower satisfaction and well-being scores as an opportunity to introduce a mindfulness program within those workgroups. Some sites either leveraged personal contacts (e.g., connecting with supervisors to increase buy-in for allowing staff time to participate in EWH) or created new contacts (e.g., in distal community-based outpatient clinics) to help with spread.

Nevertheless, there were challenges with the non-uniform spread of EWH across the organization for most sites. While some services had less opportunity to engage in activities due to work hours (e.g., nursing staff), geographic location (e.g., community-based outpatient clinics), or exposure to EWH activities (e.g., canteen, environmental management services), other services (e.g., specialty clinic, dental clinic) were simply unaware that EWH existed at their sites.

#### Communication

Many sites used technology—electronic platforms, software, media tools (e.g., Microsoft Teams, SharePoint, Facebook) and group messaging to communicate about EWH activities and connect with employees across the medical center and campuses. These tools helped build communities as employees accessed EWH resources (e.g., a nutrition channel), and through leadership direct interaction on these platforms. For instance, a medical center director wrote a weekly personal motivational message to staff. In addition, specific group messaging practices (e.g., daily email with links and announcements) helped raise awareness and interest in EWH. A participant described a routine communication practice at their site:

*“I work with our Public Affairs officer, and she’s been wonderful to work with and help us get information out in that weekly missive and then also keep us connected with opportunities that might come from other areas around the state and other communities, and how we can engage employees that work in those communities in those opportunities.”* (Site 10)

A clear and consistent message that EWH allowed employees to serve Veterans better was also an effective way to promote the program. Some senior leaders sent emails to supervisors about training opportunities and included time during the monthly town hall meetings to discuss EWH. Many sites also acknowledged the shortcomings associated with these communication tools. Some employees disliked mass emails, tuned them out, or didn’t read them consistently. Also, employees who were not added to EWH-specific group lists didn’t receive information on offered activities to participate.

In addition to technology, some sites mentioned including information about EWH as part of the new employee orientation. Other sites used alternative outreach methods such as placing printed materials on tables at employee events along with someone to talk with staff about the EWH program. One site posted flyers and other materials in clinical areas and employee shared spaces. A site used the 15-minute wait time immediately after COVID-19 vaccine injections to provide materials for a personal health inventory, schedule coaching appointments, and provide more information on EWH program activities calendar. Some sites faced challenges with these alternative methods of communication, particularly posters in clinical areas. A participant explained:

Our team very much frowns on the idea of a lot of this stuff…I think it’s an argument over beautification versus information. It’s one thing whenever we put up posters and signs, and it may be great to get this information out there but if those responsible are not coming around and taking it down afterwards, it causes [squalor] to build and so they very quickly said, no, no, no, no we’re not allowing this anymore. (Site 2)

#### Staffing

Staffing affected both the EWH implementation team and frontline staff availability to participate in activities. Most sites recognized that implementation progress was connected to EWH implementation team staffing. First, staff consistency and longer tenure of the team members helped them work more effectively. The ability to fill EWH positions with enthusiastic team members with job-specific skillsets (e.g., nurse educators, employee occupational wellness, and program support assistants) on a timely basis, was valuable. These staff were motivated and willing to put in a lot of personal effort to make the program successful. Sites where EWH was a part of their WH department mentioned that having the department fully staffed allowed for more EWH projects to engage employees, and less dependence on other departments for support. A participant explained:

Last year we got our first Whole Health [full time equivalent employee]. We have a [Patient Service Assistant] and she is really the backbone now for everything Whole Health. It’s not just Employee Whole Health, it’s Whole Health for the whole facility. (Site 6)

Nonetheless, most sites faced barriers to staffing. First, some sites were missing key EWH roles (e.g., no EWH coordinator or administrative support) due to a slow hiring process or site-imposed limitations (e.g., leadership did not approve the position). Second, many sites mentioned understaffing, either for EWH implementation team or across the medical center. For instance, EWH implementation staff had been assigned to EWH as a collateral duty (e.g., entire team working 50% time with no dedicated staff for EWH) although full-time or more dedicated positions would have been adequate. Further, loss of key program staff led to established processes in the EWH program falling apart; one site had practically stopped all activities at the time of the interviews.

As noted in the section on leadership, many sites dealing with generalized staffing shortages or burdened by staffing coverage issues discouraged participation or limited it during working hours (e.g., weekly morning Grand Rounds or noon Lunch ‘N Learn). Staffing shortages across the medical center led to staff burnout at one site.

*“I can say personally as a provider working in Whole Health for almost three years now, I’m extremely burnt out of Whole Health, just from just a lack of support and just knowing that, yes, there are people that are divinely interested in Whole Health and do talk the talk and walk the walk, but there are so few of us that we’re so dulled that our flames are, like, out.”* (Site 8)

Last, employee participation in EWH at many sites was hindered by heavy workload. Workload was a challenge to stronger partnerships (e.g., departments or service areas being understaffed, overworked, or overtasked), and limited the participation of their overworked staff (e.g., many who would benefit the most were working overtime).

#### Culture

Culture (i.e., what people at all organizational levels value and expect as being appropriate behavior) was a key factor to EWH implementation. Sites had a positive, mixed, or non-supportive culture for EWH.

Sites with a positive culture for EWH noted that self-care for employees was part of the facility culture for years prior to the implementation of EWH. At these sites, employees joined in health-conscious activities together (e.g., taking lunch breaks, taking walks to enjoy time outside). This employee-positive culture motivated employees to be “grassroot” organizers to advocate for more EWH activities. A participant at a site with EWH positive culture:

*“People are encouraged to take their lunch break and to get outside on their lunch break. We have a beautiful campus… When I would go outside to eat lunch or something on a picnic table, people were out there walking... nurses in their scrubs going for a quick walk, and so it just really is a culture at the medical center that continues to become the norm to take care of yourself and to take a break and get some exercise even if it’s a short walk, it just helps... Five years ago, you’d never see people out walking on their lunch break…So, to see that transition, I – it’s just really nice.”* (Site 7)

*S*ome sites identified having a mixed support culture, where both supportive and less supportive perspectives of EWH strongly coexisted. One example described the less supportive “old guard” vs. more supportive younger/newer staff, where some of the “old guard” belief that EWH “is not going to last at VA” dampened enthusiasm. Differences in culture were also observed across clinical areas, staff roles, and by tour of duty. For instance, night shift supervisors did not allow staff to participate during their tour of duty, while day shift supervisors did.

The facility culture was also shaped by the local state and community cultures views on health and wellness. Some sites noted that the community culture did not prioritize health (e.g., site was in a city with few sidewalks, and the region had low ranking on health behaviors). In addition, may sites brought up the cultural expectation in healthcare to provide patient care above all else, leaving little room for engagement in EWH. As a result, administrative staff could engage more in EWH activities than clinical staff. For instance:

*“A lot of our administrative staff feel a little bit more free to participate in Whole Health activities or just, getting outdoors and say going for a ten-minute walk on a break. Whereas medical or mental health units where their task of being there to provide care or in our outpatient clinics, it’s a little bit more challenging. It’s like they don’t have the same sense of freedom to be able to participate like you see in the administrative areas.”* (Site 10)

#### The impact of the COVID-19 pandemic on EWH implementation

The COVID-19 pandemic is an emergent theme from our analysis. It was widely disruptive and created challenges to EWH implementation, by imposing restrictions on activities for safety, limiting human resources available for EWH, and inducing a shift in priorities leading to a reduction of other available resources. It also had some positive aspects in some sites.

In many cases, adjusting to safety and distancing requirements translated into a complete cessation of indoor, in-person activities for EWH (e.g., yoga, healthy kitchen, and gyms and fitness centers closing), and even some outdoor activities (e.g., VA2K walks). Moreover, some sites who managed to switch from in-person activities to virtual platforms noted a decline in employees’ participation. This was particularly true for employees without access to necessary equipment at work (e.g., computer) or employees without the ‘know how’ to use new technologies (e.g., Microsoft Teams) for virtual participation.

A few sites explained that staffing resources were diverted to address the COVID-19 pandemic. One site’s entire EWH core implementation team was re-assigned to other areas to help address these challenges without protected time for managing the EWH program. At another site, certified employee instructors working in clinical areas no longer had the time to teach other employees. One site was unable to hire a new team member. Plans to enact EWH implementation were delayed, or in one instance, prevented altogether. It also placed more burden on frontline staff, causing increased workload, burnout, and turnover.

EWH implicitly became a lower priority for senior leadership across sites with the rise of the COVID-19 pandemic. A few sites noted a loss of traction for EWH. Sites were frustrated that existing resources were redirected, reduced, or made unavailable to support EWH implementation, especially audio and visual tools for sharing EWH information. Leadership at one site eliminated the use of overhead announcements to publicize EWH activities, and all Public Affairs’ regular EWH emails and promotion were halted at yet another site. One participant explained:

*“So, with COVID all that stuff had to stop in-person and it just seems like it’s been a little bit of a struggle with like upper management to allow us to have time to set up, you know, the [VA Video Connect] or Teams links, you know, to offer the classes.”* (Site 5)

Surprisingly, the COVID-19 pandemic added some facilitating elements, such as increasing the reach of the EWH program. Many sites noted how moving to a virtual platform allowed for greater employee participation in events in a way that may not have happened otherwise. Segments of the workforce previously unable to attend in-person events (e.g., employees in distant community-based outpatient clinics) were now able to join live sessions virtually. Furthermore, employees with conflicting schedules could access recorded sessions. In one participant’s words:

*“I know COVID has caused us a lot of grief but in a lot of ways it’s felt to open our eyes to other possibilities and so, … we’ve continued these Lunch and Learns. There’s no reason why we can’t continue that ‘cause employees love it.”* (Site 2)

As EWH activities became more readily available, some sites reported greater interest and inquiries into the program by employees and leadership. Supervisors understood they needed to help support their employees in the face of rising levels of burnout and stress. One site explained that the pandemic helped spur the inclusion of EWH on the network director’s performance plan.

## Discussion

Implementing employee wellness programs such as EWH, may be critical to supporting an ever-stressed healthcare workforce. Addressing workplace exposures and their consequences requires multi-level interventions. At the most macro-level, international organizations such as the WHO and ILO call for change to address workplace exposures. In this study EWH is a program of the Veterans Health Administration within the Department of Veterans affairs, a Cabinet-level department of the United States government. Ultimately changes need to be implemented in individual organizations such as the medical centers in this study. These changes are also multi-level, including leaders and managers from senior leaders to middle managers to frontline supervisors and informal champions to individual staff. This study found that simply offering wellness activities is insufficient to engage most employees and that there are multiple factors—facilitators and barriers—affecting the implementation of the EWH wellness program. Using the LET model, the evaluation also revealed the complexity of implementing an EWH program, with interactions of factors across constructs of the model, and across organizational levels.

These findings align with existing literature on organizational and employee-level barriers to the implementation of employee wellness programs in the healthcare setting — limited policy support, lack of prioritization of employee health and wellbeing, lack of leadership support, scheduling, lack of protected time, and lack of interest in activities [13–17, 32]. Findings also expand current organizational understanding by presenting more nuanced barriers (e.g., at various levels of leadership, through communication channels, and levels of staffing barriers) and organization-level contextual facilitators (alignment, integration, staffing and culture) to EWH program implementation.

The interconnectedness of many key factors, suggests the need to address implementation at three levels: (1) organization, including policies, resource allocation and prioritizing EWH, and organization-wide communication; (2) EWH activities and implementation processes including developing and offering EWH activities, communicating their availability, making them accessible to staff having different constraints; and (3) encouraging individual staff participation through champions and co-workers and removing barriers to participation such as scheduling or lack of computer access for virtual EWH activities. Leadership support and engagement on all levels is necessary to influence the other factors. For example, senior leaders are responsible primarily for resource allocation, and their communication of support and active engagement in EWH also affects culture and employee perceptions of EWH. Middle managers’ support is essential for not only allowing but also encouraging their staff’s participation in EWH.

Similarly, the impact of the COVID-19 pandemic permeated across many other factors and affected both available resources and employee engagement. Staffing shortages was underscored. In particular, as leadership priorities shifted in response to the pandemic, more responsibility was placed on fewer staff, and heavy workload prevented employees from participating in EWH activities. Ironically, EWH may have been even more important during the height of the pandemic with increasing levels of stress and burnout among providers and staff. Yet many leaders often had a different perspective and did not support EWH, and the organizational culture in some sites discouraged staff participation in EWH by making it appear to be not giving sufficient time to patients. Overall, the establishment of wellness programs in ‘normal times’ may be critical for laying the foundation for times of increasing stress in the system.

This program evaluation is not without limitations. Our findings are based on qualitative analyses and cannot be directly generalized beyond the ten participating sites who had selected EWH as the focus of their WH work. However, we spoke with 27 people to capture many perspectives likely to exist across healthcare systems, and we only reported on factors identified across at least four sites to address this limitation. As participants were key informants from EWH implementation teams, voices of employees who should benefit from the EWH programs were not directly heard. However, key informants shared anecdotes with employees’ perspectives on EWH. Phase two in this evaluation will directly capture employee perspectives on the implementation of EWH.

## Implications for policy and practice

A set of four recommendations to address EWH implementation barriers and foster EWH implementation success emerged from the analysis conducted from an organizational transformation perspective. [1] Providing “genuine leadership support” and buy-in to the EWH program from all levels of leadership was a key to program implementation. As such, it could be operationalized through direct encouragement from senior leadership to middle managers about employee participation in EWH, regular check-ins with the EWH implementation team, making EWH a part of the organizational leadership performance plans, and/or consistent emphasis on EWH from leadership at all levels. [2] With alignment challenges caused by the lack of protected time, sites suggested the establishment of explicit policies, mandates or regulations allowing employees time to participate in EWH as part of official work duties. [3] Sites noted the importance of integrating EWH into the culture of the medical center in a way that would normalize activities throughout the day (e.g., starting meetings with a mindfulness practice). Educating leaders and managers to understand that EWH is a culture change intended to create a more “whole healthy” environment could encourage employee self-care practices and build a supportive culture at both the medical center level and national VA level. [4] Sites also recognized the importance of staffing key team positions with dedicated full-time staff who knew and believed in the program being implemented.

## Conclusion

Focusing on the health and well-being of the healthcare workforce is critical to ensuring the ongoing success of our healthcare system. The VA’s focus on EWH as a key component of their efforts may serve as an exemplar for other healthcare organizations. This evaluation provides key insights into successful implementation of EWH by highlighting the importance of concerted implementation on three levels—organization, process, and employee—propitious to cultural transformation. Study findings can be informative for the rest of the VA and beyond.

## Data Availability

Data generated and analyzed during the current evaluation are not publicly available due to participants confidentiality, in compliance with institutional review board protocols for qualitative data. However, analytic methods used have been described with adequate citation for replication purposes. Questions about the dataset should be directed to David Mohr.
